# Aggressive versus Low Dose Inhibition of the Renin-Angiotensin System for the Treatment of Microalbuminuria in Type 2 Diabetic Patients: A Pilot Study

**DOI:** 10.5402/2011/696124

**Published:** 2011-10-18

**Authors:** M. B. Davidson, N. Tareen, P. Duran, V. Aguilar, M. L. Lee

**Affiliations:** Department of Internal Medicine, Charles R. Drew University, Los Angeles, CA 90059, USA

## Abstract

*Objective*. This study compares low dose versus aggressive inhibition of the renin angiotensin system (RAS) to treat microalbuminuria (MA). *Methods*. Patients with MA after a run-in period to control BP to <130/80 mm Hg with 10 mg benazepril plus other drugs and HbA1c levels to <8.0% were randomized to either continue 10 mg benazepril (*N* = 12) or to take maximal doses of benazepril plus losartan in monthly stepwise increases to achieve normoalbuminuria (*N* = 11). Because MA is associated with CVD and inflammation, carotid intima medial thickness (CIMT) and endothelial function by peripheral arterial tonometry (PAT) as surrogate indices of atherosclerosis and highly sensitive C-reactive protein (hs-CRP) to assess inflammation were measured every six months. *Results*. BP, HbA1c levels, albumin : creatinine ratios, CIMT, PAT, and hs-CRP did not differ over a mean of 12 months between the two groups. *Conclusions*. Aggressive inhibition of the RAS is unnecessary to treat MA.

## 1. Introduction

Inhibition of the renin angiotensin system (RAS) in diabetic patients with microalbuminuria (MA) retards the development of clinical proteinuria (macroalbuminuria) and increases the return to normoalbuminuria [1], even in those who are normotensive [2, 3]. MA is associated with cardiovascular disease (CVD) [4] as well as its postulated forerunners, inflammation [5], and endothelial dysfunction [5]. At least one study shows that lowering MA leads to less CVD [6]. Although to the best of our knowledge there are no studies evaluating aggressive inhibition of the RAS in type 2 diabetic patients to treat MA, many physicians progressively increase doses of angiotensin converting enzyme inhibitors (ACE-I) and angiotensin receptor blockers (ARBs) in these patients in an attempt to restore normoalbuminuria. This prospective randomized study compares the effect of aggressive versus low dose inhibition of the RAS on MA, carotid intima media thickness (CIMT) and endothelial function as surrogates for CVD [7], and highly sensitive C-reactive protein (hs-CRP) as a manifestation of inflammation.

## 2. Patients and Methods

Type 2 diabetic patients over the age of 18 years followed in the Diabetes Clinics at either Martin Luther King, Jr-Multi-Service Ambulatory Care Center, or Hubert Humphrey Comprehensive Health Center who had an albumin : creatinine ratio of 50–299 mg/g were asked to volunteer for the study. Although the accepted definition of microalbuminuria is 30 to 300 mg/g, we selected a lower limit of 50 mg/g to increase the chances that microalbuminuria would persist during the run-in period as blood pressure (BP) and glycemia were controlled since both hypertension and hyperglycemia can cause microalbuminuria in their own right [8]. Those who agreed signed an informed consent approved by the Charles R. Drew University Institutional Review Board and entered a run-in period.

All patients at screening were receiving benazepril except one who was taking lisinopril. During the run-in period, the subjects were switched to 10 mg of benazepril per day. BP, with a target of <130/80 mm Hg, was treated by the progressive addition of the following classes of drugs with escalating doses in each to a maximum before adding the next class as necessary; diuretic (hydrochlorothiazide, indapamide, or furosemide depending on the serum creatinine), beta blocker (atenolol), and dihydropyridine calcium channel blocker (amlodipine). Treatment of glycemia was intensified if necessary by following detailed treatment algorithms [9] to achieve an HbA1c level of <8.0%. Albumin : creatinine ratios were measured monthly in morning spot urine samples to ascertain that the subjects maintained MA as their BP and glycemia were controlled.

Subjects who met the BP and HbA1c goals and maintained MA were randomized into aggressive and low dose groups and followed for 18 months or until the grant ended. Albumin : creatinine ratios in morning urine samples continued to be measured monthly. Subjects in the aggressive group received increasing doses of benazepril with the addition of increasing doses of losartan if necessary to reduce the monthly albumin : creatinine ratios to <30 mg/g as follows: benazepril 10 mg to 20 mg to 40 mg once daily, plus losartan 25 to 50 to 100 mg once daily, and lastly a second 40 mg dose of benazepril at supper. If the systolic BP fell to <100 mm Hg as the ACE-I and ARB doses were increased, doses of the drugs from the other classes were back-titrated. In the low dose group, benazepril at 10 mg daily was maintained and the anti-hypertensive medications mentioned above were adjusted based only on BP, not albumin : creatinine ratios. 

All subjects had the following measurements every three months: HbA1c levels, eGFR utilizing the simplified modification of diet in renal disease (MDRD) equations [10], hs-CRP, and 24-hour ambulatory BP; every six months, CIMT [11] and peripheral arterial tonometry (PAT) measuring reactive hyperemia (RH) via finger plethysmography to evaluate endothelial function [12]. RH-PAT, which correlates significantly with posthyperemia brachial artery ultrasound measurements [13] and reflects nitric oxide generation [14], was expressed as the ratio of the pulse wave amplitude relative to the baseline and normalized to the control arm. We also measured the response to 0.4 mg sublingual nitroglycerine (NTG) given 20 minutes after cuff deflation. The NTG-PAT was expressed as the ratio of the average of the highest value and the ones just preceding and succeeding it within the next 15 minutes after cuff deflation to the baseline value before cuff inflation again normalized to the control arm.

Baseline comparisons between the two study groups were performed using a Wilcoxon rank-sum test with a 5% significance level. The evaluation of the data for each measurement over time was based on the two group repeated measures analysis of variance. To use this parametric approach, four of the variables (eGFR, CIMT, PAT, and hs-CRP) were log-transformed to achieve approximate normality. The analysis was a modified intention to treat once a subject had measurements at three months.

## 3. Results

**Figure 1:**
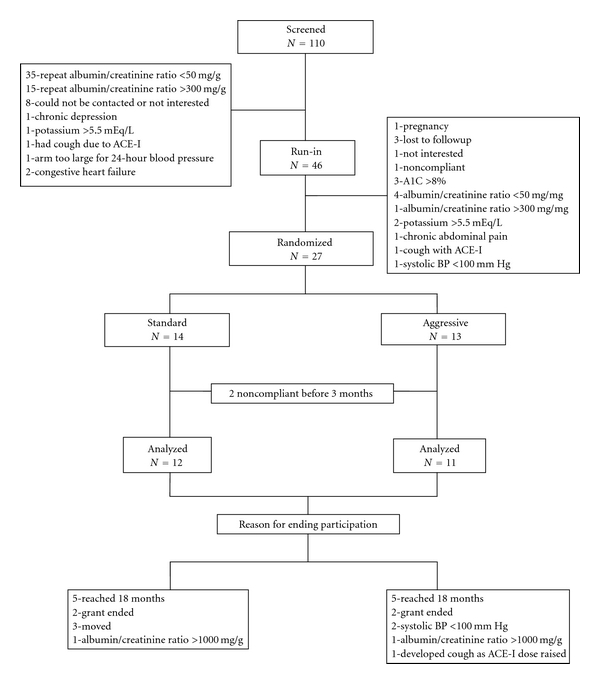
Subject flow diagram.

One hundred ten patients with MA were identified but 64 failed to reach the run-in period for the reasons shown in Figure 1. The most common reason was that on repeat urine testing for MA, 35 had values <50 mg/g and 15 had clinical proteinuria (>300 mg/g) attesting to the marked variation in day-to-day urinary albumin excretion which has a coefficient of variation of nearly 33% [15]. Forty-six entered the run-in period but 19 failed to reach randomization for reasons also shown in Figure 1. Twenty-seven patients were randomized but two in each group were noncompliant within the first three months and were dropped from the study. This left 11 subjects in the aggressive group and 12 in the standard group. The reasons for ending participation after at least three months of randomization in each group are also shown in Figure 1. The mean duration of the study in each group was 12 months.

**Figure 2:**
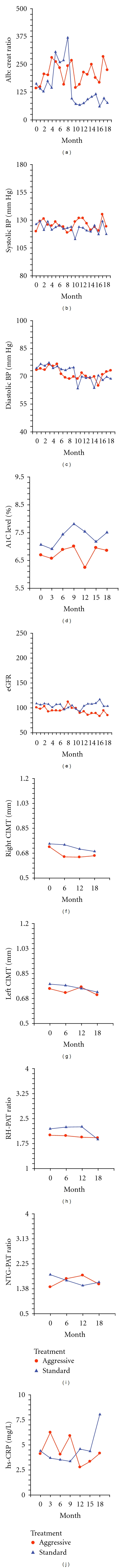
Responses after randomization; (a) albumin : creatinine ratio; (b) visit systolic BP; (c) visit diastolic BP; (d) HbA1c level; (e) estimated glomerular filtration rate; (f) right CIMT; (g) left CIMT; (h) reactive hyperemia-induced peripheral arterial tonometry; (i) nitroglycerine-induced peripheral arterial tonometry; (j) highly sensitive C-reactive protein. The number of subjects followed at each three month interval in the aggressive- and low-dose groups, respectively, were three months-11 and 12; six months-9 and 11, nine months-8 and 9, 12 months 6 and 6; 15 months-6 and 6; and 18 months-5 and 5. Standard : low dose. Data in graphs (a), (b), (c), and (e) were measured monthly but shown bimonthly for ease of presentation.

There were no significant differences at randomization between the two groups at randomization (Table 1) except that in the aggressive group the duration of diabetes was significantly less than in the low dose group. Medications at randomization and at the conclusion of the study are shown in Table 2. In the aggressive group at the final visit, seven were taking 80 mg benazepril, four 40 mg benazepril, eight 100 mg losartan, and two 50 mg losartan. As expected, because the subjects were brought under control to HbA1c levels <8.0% before randomization, there was little change in the anti-hyperglycemic medications during the study. Figure 2 shows the responses in the two groups over time. There were no differences in albumin : creatinine ratios, visit systolic and diastolic BPs, HbA1c levels, eGFR, CIMTs, or RH-PAT and NTG-PAT. Twenty-four-hour BPs, whether analyzed over the entire period or in daytime and nighttime 12-hour periods, showed the same results, that is, no difference in systolic or diastolic BPs (data not shown). One subject in each group returned to normoalbuminuria, three subjects in the aggressive group and one in the low dose group developed clinical proteinuria leading to one in each group being discontinued because their albumin : creatinine ratios exceeded one gram in two consecutive urine samples after six months.

## 4. Discussion

The effect of aggressive inhibition of the RAS system per se on microalbuminuria could be reliably evaluated because glycemia and BP were controlled before randomization and throughout the study (Figures 2(b), 2(c), and 2(d)). One might have expected that the shorter duration of diabetes in the aggressively treated group might have favored the return of MA to normoalbuminuria but that was not the case. Aggressive inhibition of the RAS had no beneficial effect on MA compared to low dose inhibition (Figure 2(a)). Although an obvious limitation of the study is the small number of subjects, the albuminuria responses make it very unlikely that more subjects or an extended period of observation would show a beneficial effect of high doses of an ACE-I plus an ARB because MA was worsening in the aggressive group after 10 months (Figure 2(a)). These data extend the results of the IMPROVE trial in which a submaximal dose of an ACE-I plus a maximal dose of an ARB was no more effective in reducing MA than the submaximal dose of the ACE-I alone [16]. They are also consistent with results in normotensive type 1 diabetic patients with MA in which there was no difference in progression to macroalbuminuria over two years in those receiving 1.5 mg or 5.0 mg of ramipril [17].

The present results do not invalidate the overwhelming evidence that inhibition of the RAS retards the development of overt nephropathy in patients with MA [1] because these subjects had already been treated with varying doses of an ACE-I for many years before enrolling in the study. It does show that more aggressive inhibition is ineffective. Thus, physicians caring for patients with MA need only provide low doses of an ACE-I or an ARB for treating MA and control both BP (with other antihypertensive medications if they desire) and glycemia for possible further reductions in MA.

## Figures and Tables

**Table 1:** Characteristics (mean ± SD where applicable) at randomization.

	Low dose	Aggressive	*P* value
Number of subjects	12	11	
Age (years)	54.4 ± 6.7	51.6 ± 5.9	NS^a^
Males/females	5/7	7/4	
Duration of diabetes (years)	12.8 ± 5.4	7.1±3.1	<0.01
Race/ethnicity			
Latino	10	10	
African American	2	0	
Caucasian	0	1	
Alb/creat (mg/g)	162.7 ± 94.0	142.2 ± 72.4	NS
Visit systolic BP (mm Hg)	126.2 ± 7.1	119.7 ± 10.6	NS
Visit diastolic BP (mm Hg)	74.3 ± 10.8	73.4 ± 5.5	NS
24-Hour systolic BP (mm Hg)	125.9 ± 6.8	120.9 ± 10.3	NS
24-Hour diastolic BP (mm Hg)	73.7 ± 8.7	70.7 ± 4.3	NS
12-Hour AM systolic BP (mm Hg)	129.0 ± 6.5	122.0 ± 10.2	NS
12-Hour AM diastolic BP (mm Hg)	75.9 ± 8.7	72.2 ± 5.0	NS
12-Hour PM systolic BP (mm Hg)	123.1 ± 9.1	119.4 ± 10.6	NS
12-Hour PM diastolic BP (mm Hg)	71.7 ± 10.0	69.2 ± 4.8	NS
Body mass index	32.2 ± 5.9	33.7 ± 3.9	NS
HbA1c (%)	7.1 ± 0.6	6.7 ± 0.6	NS
eGFR (mL/min/1.73 m^2^)^b^	108.7 ± 21.6	100.5 ± 33.7	NS
Right CIMT (mm)^b^	0.74 ± 0.09	0.72 ± 0.17	NS
Left CIMT (mm)^b^	0.78 ± 0.16	0.74 ± 0.16	NS
	(*N* = 11)	(*N* = 9)	
PAT-Hyperemia-induced (fold increase)^b^	2.2 ± 0.5	2.0 ± 0.7	NS
PAT-NTG-induced (fold increase)^b^	1.9 ± 0.8	1.4 ± 0.6	NS
hs-C-reactive protein^b^ (mg/L)^b^	4.3 ± 3.6	4.1 ± 3.1	NS

^a^ *P* > 0.05; ^b^log transformed; BP: blood pressure; eGFR: estimated glomerular filtration rate; HbA1c: hemoglobin A1c; CIMT: carotid intima medial thickness; PAT: peripheral arterial tonometry; hs-CRP: highly sensitive C-reactive protein.

**Table 2:** Medications.

Medications	Low dose		Aggressive	
	(*N* = 12)		(*N* = 11)	
	Randomization	Final	Randomization	Final
*Anti-hypertensives*				
Benazepril	12	12	11	11
Losartan	0	0	0	10
Diuretic^a^	6	10	7	3
Amlodipine	3	4	2	4
Atenolol	0	0	4	4
*Anti-hyperglycemics*				
Metformin	12	12	11	11
Sulfonylurea^b^	7	7	8	8
Pioglitazone	5	5	4	3
Insulin	6	7	4	4

^a^Hydrochlorothiazide, indapamide, or furosemide; ^b^glyburide, glipizide, or glimepiride.

## Abbreviations

ACE-I: Angiotensin converting enzyme inhibitor
ARB: Angiotensin receptor blocker
BP: Blood pressure
CIMT: Carotid intima medial thickness
CVD: Cardiovascular disease
eGFR: Estimated glomerular filtration rate
Hb: Hemoglobin
hs-CRP: Highly sensitive C-reactive protein
MA: Microalbuminuria
MDRD: Modification of diet in renal disease
NTG: Nitroglycerin
PAT: Peripheral arterial tonometry
RH: Reactive hyperaemia.
